# Extracranial-intracranial bypass surgery for occlusive atherosclerotic disease of the anterior cerebral circulation: protocol for a systematic review and meta-analysis

**DOI:** 10.1186/s13643-020-01325-6

**Published:** 2020-04-02

**Authors:** Xuesong Bai, Yao Feng, Kun Yang, Tao Wang, Jichang Luo, Xue Wang, Feng Ling, Yan Ma, Liqun Jiao

**Affiliations:** 1 Department of Neurosurgery, Xuanwu Hospital, Capital Medical University, No. 45 Changchun Street, Beijing, 100053 China; 2 Department of Evidence-Based Medicine, Xuanwu Hospital, Capital Medical University, No. 45 Changchun Street, Beijing, China; 3 Medical Library, Xuanwu Hospital, Capital Medical University, No. 45 Changchun Street, Beijing, China

**Keywords:** Occlusive atherosclerotic disease, Anterior cerebral circulation, Extracranial-intracranial bypass surgery, Best medical therapy, Meta-analysis

## Abstract

**Background:**

Occlusive atherosclerotic disease of the anterior cerebral circulation is one of the most common causes of anterior circulation ischemia and stroke. Treatment options include medical therapies (including antiplatelet use, blood pressure control, lipid reduction, and lifestyle modification) and extracranial-intracranial bypass surgery (such as superficial temporal artery-middle cerebral artery bypass). However, the optimal treatment remains unclear. The objective of this study will be to compare the efficacy of and extracranial-intracranial bypass surgery with that of other medical therapy in adult patients with occlusive atherosclerotic disease of the anterior cerebral circulation.

**Methods:**

This is the study protocol for a systematic review. We will search MEDLINE, EMBASE, Web of Science, and the Cochrane Library (from January 1980 onwards). We will include randomized controlled trials, quasi-experimental studies (non-randomized, interrupted time series), and observational studies (e.g., cohort studies and case-control studies), examining the efficacy of extracranial-intracranial bypass surgery compared to other treatments for adult patients with occlusive atherosclerotic disease of anterior cerebral circulation. Two team members will independently screen all citations, full-text articles, and abstract data. Potential conflicts will be resolved through discussion. The primary outcome will include stroke or death. The secondary outcomes will include intracranial hemorrhage, transient ischemic attack, and myocardial infarction. The study methodological quality (or bias) will be appraised using appropriate tools. If feasible, we will conduct random effects meta-analysis. Additional analyses will be conducted to explore the potential sources of heterogeneity (e.g., study design, geographical location, or risk of bias).

**Discussion:**

This review will evaluate the evidence on the efficacy of extracranial-intracranial bypass surgery for adult patients with occlusive atherosclerotic disease of the anterior cerebral circulation. We anticipate that our findings will be of interest to patients, their families, caregivers, healthcare professionals, and in making optimal treatment selection. Implications for future clinical and epidemiological research will be discussed.

**Systematic review registration:**

PROSPERO CRD42018105513

## Background

Stroke is a significant global health burden cause [1–3]. Nearly 20% of patients with anterior circulation ischemia may have carotid artery occlusion [4–6]. This disease could also increase the risk of death among middle-aged and older patients [7]. Atherosclerotic arterial occlusion could be attributed to several risk factors, including hypertension [8], diabetes [8–10], dyslipidemia [8, 11], smoking [8, 9, 12], homocysteine [13], chronic reactive protein (CRP) [14], sex [7], and race [15, 16]. Predominant symptoms and signs may be variable. Visual, motor, and sensory deficits are the most common symptoms [8]. Impaired cerebral hemodynamics due to occlusive cerebrovascular lesion may increase the risk of subsequent strokes [5, 17]. Furthermore, patients with increased oxygen extraction fraction (OEF) had higher risk of future symptom onset than patients with normal OEF [18].

The best medical therapy (BMT) could reduce the risk of vascular events through the use of synthetic drugs involving blood pressure control, anti-antiplatelet aggregation, and hyperlipidemia management, as well as through lifestyle modification [2, 19, 20]. BMT focuses on the continuous and long-term use of synthetic drugs to prevent future stroke [2, 21]. However, some of these patients, especially those who are hemodynamically compromised, showed unsatisfactory outcomes after receiving BMT [22]. It has been reported that 5 to 8% of these patients may have an ipsilateral ischemic stroke during the first 2 years [5, 23, 24]. At the same time, revascularization by extracranial-intracranial (EC-IC) bypass surgery was firstly performed by Yasargil [25]. It was then utilized to treat occlusive cerebrovascular disease [18, 26]. The bypass surgery is mainly performed by connecting the superficial temporal artery (STA) to the middle cerebral artery (MCA). The surgery could improve regional cerebral blood volume as well as OEF [18]. Therefore, EC-IC bypass surgery may be effective in stroke prevention [22, 27–30], but was hypothesized to benefit only hemodynamically compromised patients [17, 21]. In addition, a high risk of hemorrhage stroke was observed in the early postoperative period, which may obscure the true benefit of revascularization of the ischemic brain [31].

The best treatment for occlusive atherosclerotic disease of the anterior cerebral circulation remains controversial. Firstly, the medical treatment provided in these patients had unsatisfactory outcomes, and the EC/IC Bypass Study reported disappointing results of the bypass surgery [32]. Secondly, it was hypothesized that bypass surgery would be more suitable for hemodynamically compromised patients [17]. However, two subsequent randomized controlled trials (RCTs), including the Japanese EC-IC bypass trial (JET) [33] and Carotid and Middle cerebral artery Occlusion Surgery Study (CMOSS) [34, 35] did not show that bypass surgery was superior to BMT in terms of stroke prevention. Therefore, whether bypass surgery is superior to medical therapy remains unknown. To our knowledge, only one systemic review and meta-analysis comparing the two treatment modalities were published in 2010, [36] and several important clinical studies have been published ever since [22, 34, 35, 37, 38].

The objective of this study will be to compare the efficacy of and extracranial-intracranial bypass surgery with that of other medical therapy in adult patients with occlusive atherosclerotic disease of the anterior cerebral circulation.

## Methods

The present protocol has been registered within the PROSPERO database (registration number CRD42018105513) and is being reported in accordance with the reporting guidance provided in the Preferred Reporting Items for Systematic Reviews and Meta-Analyses Protocols (PRISMA-P) statement [39] (see checklist in Additional file 1). This systematic review will be conducted using the methodological guidance in the Cochrane Handbook for Systematic Reviews of Interventions [40]. Any amendments made to this protocol when conducting the study will be outlined and reported in PROSPERO and the final manuscript

### Information sources and search strategy

The primary source of literature will be a structured search of major electronic databases (from January 1980 onwards): PubMed/MEDLINE, EMBASE, Web of Science, and the Cochrane Library. The secondary source of potentially relevant material will be a search of the grey or difficult to locate literature, including clinical trials registers (such as ClinicalTrials.gov). We will perform hand searching of the reference lists of included studies, relevant reviews, national clinical practice guidelines, or other relevant documents. Content experts and authors who are prolific in the field will be contacted. The literature searches will be designed and conducted by the review team which includes two experienced health information specialists. The search will include a broad range of terms and keywords related to “anterior cerebal artery,” “occlusion,” and “bypass surgery.” A draft search strategy for PubMed/MEDLINE is provided in Additional file 2**.**

### Study selection

The Population, Intervention, Comparison, Outcome (PICO) model will be used in determining the specific criteria for selecting studies [41].

#### Participants

The study participants will include patients aged 18 years or over and experienced ischemic stroke caused by occlusive atherosclerotic disease (verified by angiography) of the anterior cerebral circulation. Moreover, they should include those who underwent EC-IC bypass surgery or received BMT. The location of stenosis should be in the internal carotid artery (ICA) or subsequent branches. Meanwhile, patients with stenosis due to nonatherosclerotic etiologies, including arterial dissection, moyamoya disease, vasculitis disease, radiation-induced vasculopathy, fibromuscular dysplasia, sickle cell disease, neurofibromatosis, suspected vasospastic process, and suspected recanalized embolus, will be excluded [36].

#### Type of intervention

We will examine studies investigating EC-IC bypass surgery for atherosclerotic occlusion of the anterior cerebral circulation. The EC-IC bypass must be the primary purpose of the intervention. It includes low-flow bypass such as superficial temporal artery-middle cerebral artery (STA-MCA) bypass and high-flow bypass such as external carotid artery-radial artery-middle cerebral artery (ECA-RA-MCA) bypass.

#### Comparator

The comparator will be BMT, which aims to control blood pressure control, inhibit platelet aggregation, manage hyperlipidemia, and modify lifestyle [34, 42]

#### Outcome

The following primary and secondary outcomes will be evaluated.

##### Primary outcomes

Stroke or death during (1) perioperative period and (2) long-term follow-up (e.g., 1 and 2 years).

##### Secondary outcomes

Intracranial hemorrhage, transient ischemic attacks, and myocardial infarction (1) during perioperative period and (2) long-term follow-up (e.g., 1 and 2 years)

#### Studies design

We will include various types of studies, including RCTs, quasi-experimental studies (e.g., non-randomized trials and interrupted time series), and observational studies (e.g., cohort studies and case-control studies). Observational studies will be included to observe the occurrence of rare complications with a clinical value and to minimize type II error, which is due to lack of statistical power of RCTs [43]. Case series or reports and experimental studies on animals will be excluded.

### Data extraction, synthesis and analysis

#### Data extraction

The EndNote X7 software will be used to manage literature searches. Potentially eligible studies will be identified by two independent authors by reviewing the titles and abstracts. Then, in accordance with the above inclusion criteria, two authors will assess all potentially eligible studies by screening of the full text. Consulting a third review author will be necessary if discrepancies are found. The reasons for excluding the studies from the review will be provided. Information about ongoing trials and duplication will also be provided. The process of selecting relevant studies process will be reported by using a PRISMA flow chart.

Data extraction will be performed by two independent reviewers. A standardized electronic form for data extraction will be used. We will extract all relevant data of interest from the included studies for comparison, such as (1) study and patients’ characteristics, (2) group by intervention type, (3) detailed information of surgery techniques and medical treatment plan, and (4) primary and secondary outcomes with observation time points. If there were disagreements on data extraction between the two reviewers, a team group discussion would be organized for a final decision.

Dichotomous data will be documented as frequencies, while continuous data will be documented as mean value and standard deviation, along with the number of patients in each group. When encountering a necessary outcome that is inaccessible for direct data extraction, we will try to contact the authors by sending an email. If we do not receive a response from the author, a second email will be sent. We will exclude the study if there will be no responses to both emails, and the condition will be documented in the PRISMA flow chart.

#### Assessment of risk of bias

Two reviewers will independently assess the risk of bias for each study with risk of bias 2.0 tool for RCTs and quasi-experimental studies [40] and the Newcastle-Ottawa Scale (NOS, Additional file 3) for observational studies at study level [44, 45]. A third reviewer will help resolve any conflict through discussion. We will assess the risk of bias of RCTs and quasi-experimental studies according to the following seven domains:

•Random sequence generation

•Allocation concealment

•Blinding of participants and personnel

•Blinding of outcome assessment

•Incomplete outcome data

•Selective outcome report

•Other possible bias

We will assess the risk of bias of observational studies through three domains including selection, compatibility, and exposure. We will grade the risk of bias as high, low, or unclear for each domain. We will also provide information from the study report together with a justification for our judgment in the “Risk of bias” tables.

#### Data synthesis and analysis

We will synthetize primary studies to explore heterogeneity descriptively rather than statistically such as structured narratives, summary tables, and measures of treatment effects. Then, a meta-analysis for quantitatively synthesis on one specific result will be performed when at least two suitable studies are included. Since clinical and epidemiological heterogeneity is expected a priori, meta-analyses will be conducted using the random effects model when data are appropriate. The random effects model assumes the treatment effects follow a normal distribution, considering both within-study and between-study variation. The analysis will be conducted using the Review Manager software [46]. When a meta-analysis was not feasible due to an insufficient number of studies, we provided a narrative description of the study results. We will create a “Summary of findings” table using the following outcomes: stroke or death (during perioperative period and long-term follow-up), intracranial hemorrhage, transient ischemic attacks, and myocardial infarction (during perioperative period and long-term follow-up). Continuous outcomes will be expressed as standardized mean difference (SMD) along with its 95% confidence interval (CI). Dichotomous outcomes will be expressed as relative risk (RR) along with its 95% CI. Forest plots will be used to visualize pooled estimates and the extent of heterogeneity among studies. Heterogeneity will be assessed using the *I*^2^ test. We will quantify statistical heterogeneity by estimating the variance between studies using *I*^2^ statistic. The *I*^2^ statistic is the proportion of variation in prevalence estimates that is due to genuine variation in prevalence rather than sampling (random) error. *I*^2^ statistic ranges between 0 and 100%, with values of 0–25% and 75–100%, respectively, taken to indicate low and considerable heterogeneity. We will also report Tau^2^ and Cochran *Q* test with a *P* value of < 0.05 considered statistically heterogeneity. When heterogeneity was found, we will conduct a subgroup analysis of the following variables if the data were sufficient, such as study design, geographical location, or risk of bias.

#### Meta-biases assessment

Two independent team members will also examine possible meta-biases, including publication bias, selective outcome reporting, and dual co-authorship [47, 48]. Handling of heterogeneity for meta-analyses and other potential sources of bias will be described.

#### Confidence in cumulative evidence

The quality of a body of evidence will be evaluated in according to the principle of the Grading of Recommendations Assessment, Development, and Evaluation (GRADE) system [45] through the five considerations (study limitations, consistency of effect, imprecision, indirectness, and publication bias). We will use methods and recommendations described of the Cochrane Handbook for Systematic Reviews of Interventions and the GRADEpro GDT software.

## Discussion

The planned systematic review and meta-analysis may shed light on the strategy selection when treating patients with occlusive atherosclerotic disease of the anterior cerebral circulation, especially those who are hemodynamically compromised. As many recent studies recommended that bypass surgery may only be suitable for patients with impaired cerebral hemodynamics, an update of clinical evidence of comparing the two modalities is necessary. We anticipate that our findings will be of interest to patients, their families, caregivers, and healthcare professionals in making optimal treatment selection. There are several strengths and limitations of our planned systematic review methods. We will comprehensively evaluate both randomized and epidemiological data characteristics, including detailed information of interventions and primary and secondary outcomes. We hope that we will identify knowledge gaps to be filled by new researches. On this regard, implications for future research will be discussed in the final manuscript. A key challenge is that based on knowledge of the review team, we anticipate identifying studies using different study designs, populations, contexts, and with a variable quality of reporting methods and results. So, we will keep the stringent rules in the assessment of study risk bias and extraction of data to minimize the unfavorable impact on outcomes comparison by observational study inclusion. Results will be disseminated through conference presentations and publication in a peer-reviewed journal.

## Supplementary information

**Additional file 1.** PRISMA-P (Preferred Reporting Items for Systematic review and Meta-Analysis Protocols) 2015 checklist: recommended items to address in a systematic review protocol.

**Additional file 2.** Medline search strategy.

**Additional file 3.** Newcastle - Ottawa Quality assessment scale case control studies.

## Data Availability

Data sharing is not applicable to this article as no datasets were generated or analyzed during the current study.

## Abbreviations

CRP: Chronic reactive protein
OEF: Oxygen extraction fraction
BMT: Best medical therapy
EC-IC: Extracranial-intracranial
STA: Superficial temporal artery
MCA: Middle cerebral artery
RCT: Randomized controlled trial
JET: Japanese EC-IC bypass trial
CMOSS: Carotid and Middle cerebral artery Occlusion Surgery Study
PROSPERO: International prospective register of systematic reviews
PRISMA: Preferred Reporting Items for Systematic Reviews and Meta-Analyses
PICO: Population, Intervention, Comparison, Outcome
STA-MCA: Superficial temporal artery-middle cerebral artery
ECA-RA-MCA: External carotid artery-radial artery-middle cerebral artery
SMD: Standardized mean difference
RR: Relative risk
CI: Confidence interval
