# Supplementary material for: Extracranial-intracranial bypass surgery for occlusive atherosclerotic disease of the anterior cerebral circulation: protocol for a systematic review and meta-analysis
Source: Syst Rev. 2020 Apr 2;9:70. doi: 10.1186/s13643-020-01325-6 (PMC7118989; doi:10.1186/s13643-020-01325-6)
Supplement: Supplementary file 1 — Additional file 1. PRISMA-P (Preferred Reporting Items for Systematic review and Meta-Analysis Protocols) 2015 checklist: recommended items to address in a systematic review protocol. [file 13643_2020_1325_MOESM1_ESM.pdf]

Additional file 1. PRISMA-P (Preferred Reporting Items for Systematic review and Meta-Analysis Protocols) 2015 checklist: recommended items to address in a systematic review protocol*

Section and topic	Item No	Checklist item	Check results
**ADMINISTRATIVE INFORMATION**			
Title:			P1, L1-3
Identification	1a	Identify the report as a protocol of a systematic review	Yes
Update	1b	If the protocol is for an update of a previous systematic review, identify as such	Yes
Registration	2	If registered, provide the name of the registry (such as PROSPERO) and registration number	Yes<br>P2, L30
Authors:			
Contact	3a	Provide name, institutional affiliation, e-mail address of all protocol authors; provide physical mailing address of corresponding author	Yes<br>P1, L8-18
Contributions	3b	Describe contributions of protocol authors and identify the guarantor of the review	Yes<br>P7, L8-13
Amendments	4	If the protocol represents an amendment of a previously completed or published protocol, identify as such and list changes; otherwise, state plan for documenting important protocol amendments	Yes
Support:			P8, L1-4
Sources	5a	Indicate sources of financial or other support for the review	Yes
Sponsor	5b	Provide name for the review funder and/or sponsor	Yes

Role of sponsor or funder	5c	Describe roles of funder(s), sponsor(s), and/or institution(s), if any, in developing the protocol	Yes
**INTRODUCTION**			
Rationale	6	Describe the rationale for the review in the context of what is already known	Yes<br>P3, L2-39
Objectives	7	Provide an explicit statement of the question(s) the review will address with reference to participants, interventions, comparators, and outcomes (PICO)	Yes<br>P4, L1-3
**METHODS**			
Eligibility criteria	8	Specify the study characteristics (such as PICO, study design, setting, time frame) and report characteristics (such as years considered, language, publication status) to be used as criteria for eligibility for the review	Yes<br>P4, L28-P5, L30
Information sources	9	Describe all intended information sources (such as electronic databases, contact with study authors, trial registers or other grey literature sources) with planned dates of coverage	Yes<br>P4, L15-27
Search strategy	10	Present draft of search strategy to be used for at least one electronic database, including planned limits, such that it could be repeated	Yes<br>Additional file 2
Study records:			
Data management	11a	Describe the mechanism(s) that will be used to manage records and data throughout the review	Yes<br>P6, L4-5
Selection process	11b	State the process that will be used for selecting studies (such as two independent reviewers) through each phase of the review (that is, screening, eligibility and inclusion in meta-analysis)	Yes<br>P6, L6-12
Data collection process	11c	Describe planned method of extracting data from reports (such as piloting forms, done independently, in duplicate), any processes for obtaining and confirming data from investigators	Yes<br>P6, L13-19

Data items	12	List and define all variables for which data will be sought (such as PICO items, funding sources), any pre-planned data assumptions and simplifications	Yes<br>P6, L6-12
Outcomes and prioritization	13	List and define all outcomes for which data will be sought, including prioritization of main and additional outcomes, with rationale	Yes<br>P5, L17-23
Risk of bias in individual studies	14	Describe anticipated methods for assessing risk of bias of individual studies, including whether this will be done at the outcome or study level, or both; state how this information will be used in data synthesis	Yes<br>P6, L20-P7, L2
Data synthesis	15a	Describe criteria under which study data will be quantitatively synthesised	Yes<br>P7, L4-7
	15b	If data are appropriate for quantitative synthesis, describe planned summary measures, methods of handling data and methods of combining data from studies, including any planned exploration of consistency (such as I^2, Kendall's τ)	Yes<br>P7, L13-26
	15c	Describe any proposed additional analyses (such as sensitivity or subgroup analyses, meta-regression)	Yes<br>P7, L27-29
	15d	If quantitative synthesis is not appropriate, describe the type of summary planned	Yes<br>P7, L12-14
Meta-bias(es)	16	Specify any planned assessment of meta-bias(es) (such as publication bias across studies, selective reporting within studies)	Yes<br>P7, L32-34
Confidence in cumulative evidence	17	Describe how the strength of the body of evidence will be assessed (such as GRADE)	Yes<br>P7, L37-P8, L4

*** It is strongly recommended that this checklist be read in conjunction with the PRISMA-P Explanation and Elaboration (cite when available) for important clarification on the items. Amendments to a review protocol should be tracked and dated.** **The copyright for PRISMA-P (including checklist) is held by the PRISMA-P Group and is distributed under a Creative Commons Attribution Licence 4.0.**

From: Shamseer L, Moher D, Clarke M, Ghersi D, Liberati A, Petticrew M, Shekelle P, Stewart L, PRISMA-P Group. Preferred reporting items for systematic review and meta-analysis protocols (PRISMA-P) 2015: elaboration and explanation. BMJ. 2015 Jan 2;349(jan02 1):g7647.
