# Supplementary material for: Extracranial-intracranial bypass surgery for occlusive atherosclerotic disease of the anterior cerebral circulation: protocol for a systematic review and meta-analysis
Source: Syst Rev. 2020 Apr 2;9:70. doi: 10.1186/s13643-020-01325-6 (PMC7118989; doi:10.1186/s13643-020-01325-6)
Supplement: Supplementary file 2 — Additional file 2. Medline search strategy. [file 13643_2020_1325_MOESM2_ESM.pdf]

Supplement 2. Medline search strategy

Search step	Search terms
#1	"Anterior Cerebral Artery" [Mesh] OR "Middle Cerebral Artery" [Mesh] OR "Carotid Arteries" [Mesh] OR "Carotid Artery, Internal" [Mesh] OR Anterior Cerebral Arteries [All Fields] OR Arteries, Anterior Cerebral [All Fields] OR Artery, Anterior Cerebral [All Fields] OR Cerebral Arteries, Anterior [All Fields] OR Cerebral Artery, Anterior [All Fields] OR Arteries, Middle Cerebral [All Fields] OR Artery, Middle Cerebral [All Fields] OR Cerebral Artery, Middle [All Fields] OR Middle Cerebral Arteries [All Fields] OR Cerebral Arteries, Middle [All Fields] OR Arteries, Carotid [All Fields] OR Carotid Artery [All Fields] OR Artery, Carotid [All Fields] OR Arteries, Internal Carotid [All Fields] OR Artery, Internal Carotid [All Fields] OR Carotid Arteries, Internal [All Fields] OR Internal Carotid Arteries [All Fields] OR Internal Carotid Artery [All Fields]
#2	"Cerebrovascular Disorders" [Mesh] OR Cerebrovascular Occlusion [All Fields] OR Occlusions, Cerebrovascular [All Fields] OR Occlusion, Cerebrovascular [All Fields] OR Cerebrovascular Occlusions [All Fields] OR Cerebrovascular Diseases [All Fields] OR Diseases, Cerebrovascular [All Fields] OR Disease, Cerebrovascular [All Fields] OR Cerebrovascular Disease [All Fields] OR occlu* [All Fields] OR obstruct* [All Fields]
#3	#1 AND #2
#4	"Cerebral Revascularization" [Mesh] OR Extracranial-Intracranial Arterial Bypass [All Fields] OR Arterial Bypass, Extracranial-Intracranial [All Fields] OR Arterial Bypasses, Extracranial-Intracranial [All Fields] OR Bypass, Extracranial-Intracranial Arterial [All Fields] OR Bypasses, Extracranial-Intracranial Arterial [All Fields] OR Extracranial Intracranial Arterial Bypass [All Fields] OR Extracranial-Intracranial Arterial Bypasses [All Fields] OR EC-IC Arterial Bypass [All Fields] OR Arterial Bypass, EC-IC [All Fields] OR Bypasses, EC-IC Arterial [All Fields] OR Bypass, EC-IC Arterial [All Fields] OR EC IC Arterial Bypass [All Fields] OR EC IC Arterial Bypass [All Fields] OR STA-MCA Bypass [All Fields] OR Bypass, STA-MCA [All Fields] OR Bypasses, STA-MCA [All Fields] OR STA MCA Bypass [All Fields] OR STA-MCA Bypasses [All Fields]
#5	#3 AND #4
